# Changes in Incidence of Notifiable Infectious Diseases in China Under the Prevention and Control Measures of COVID-19

**DOI:** 10.3389/fpubh.2021.728768

**Published:** 2021-10-15

**Authors:** Bizhen Chen, Meiling Wang, Xun Huang, Maokun Xie, Liting Pan, Huiwen Liu, Zhenguo Liu, Pengcheng Zhou

**Affiliations:** ^1^Department of Healthcare-Associated Infection Management, The Second Affiliated Hospital of Fujian University of Traditional Chinese Medicine, Fuzhou, China; ^2^Department of Infectious Diseases/Infection Control Center, The Third Xiangya Hospital of Central South University, Changsha, China; ^3^School of Nursing, Fujian University of Traditional Chinese Medicine, Fuzhou, China; ^4^Infection Control Center, Xiangya Hospital of Central South University, Changsha, China

**Keywords:** COVID-19, notifiable infectious diseases, public health interventions, incidence, disease transmission

## Abstract

**Aim:** The aim of this study was to analyze the changes in incidence of notifiable infectious diseases in China under the prevention and control measures of COVID-19.

**Methods:** Using descriptive epidemiological methods, data were collected from the official website of the Health Commission of the People's Republic of China, and the prevalence characteristics of notifiable infectious diseases in the country in 2020 were analyzed and compared with the historical data in 2019. Monthly reporting data on influenza and tuberculosis from 2015 to 2019 were also collected.

**Results:** Except for COVID-19, the total number of notifiable infectious diseases cases in 2020 was 6,366,176, a decrease of 41.38% year-on-year compared with 2019. Category B and C notifiable infectious diseases decreased by 14.84 and 54.98% year-on-year, respectively (*P* < 0.01). The top three incidence rates were influenza (87.63 cases/100,000), hepatitis B (81.36 cases/100,000) and other infectious diarrhea (76.33 cases/100,000). Three types of diseases with the largest decline were influenza (−2,280,502 cases), hand-foot-mouth disease (−1,174,588 cases), and other infectious diarrhea diseases (−275,746 cases). Compared with 2019, respiratory infectious diseases were reported to be in the largest decline in 2020, followed by intestinal infectious diseases, blood-borne and sexually transmitted diseases, natural foci, and insect-borne infectious diseases. The monthly reported incidences of influenza and tuberculosis in 2020 were lower than the average of the previous 5 years.

**Conclusion:** In 2020, the incidence of most notifiable infectious diseases in China showed a downward trend, non-pharmaceutical interventions (NPIs)such as the wearing of masks, frequent hand-washing, more ventilation, less gathering, etc, played an positive role in the prevention and control of respiratory and intestinal infectious diseases. The various public health intervention strategies and measures adopted by China to contain COVID-19 can provide a reference for the prevention and control of infectious diseases in other countries.

## Introduction

**Figure 1:**
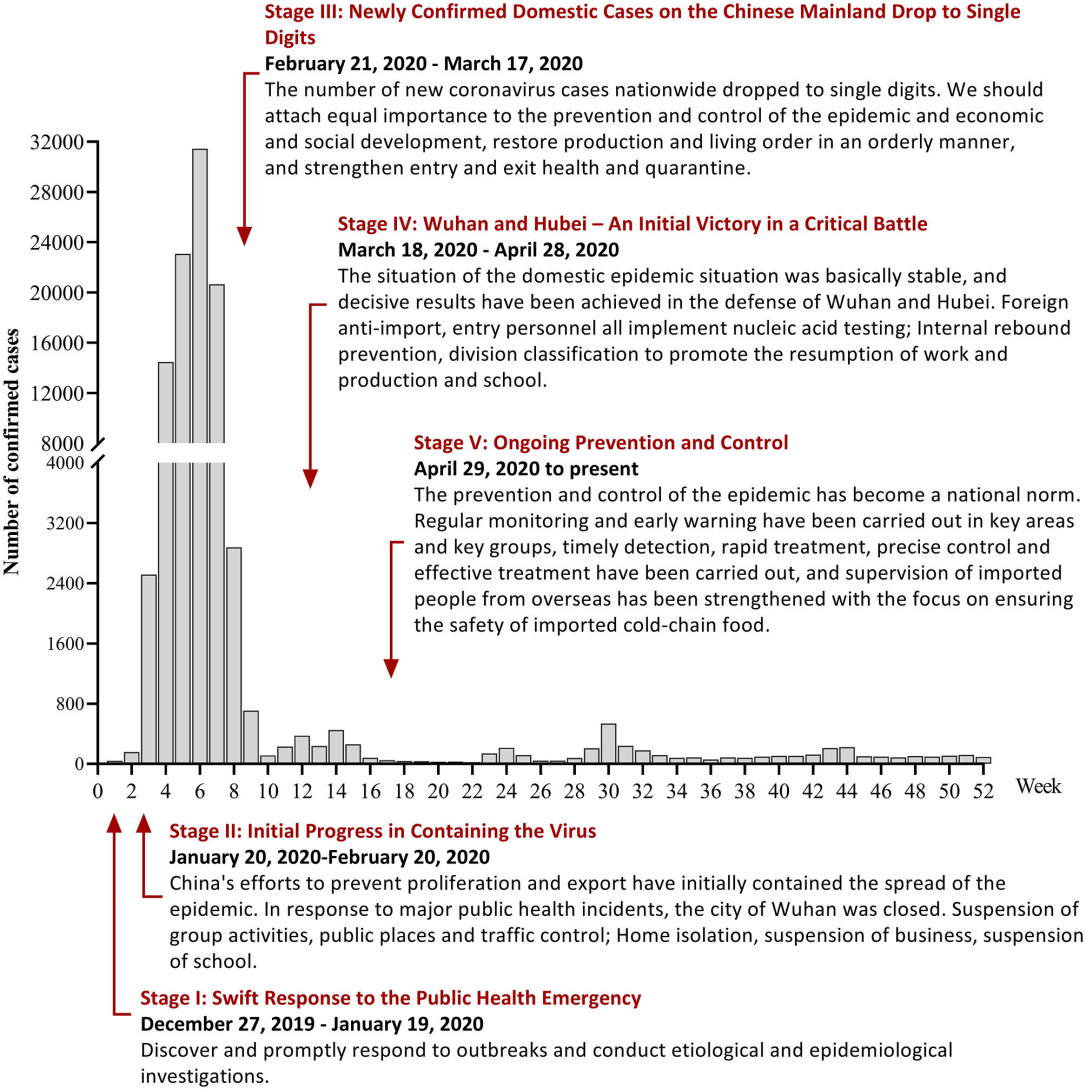
Incidence and public interventions of COVID-19 in China in 2020.

Since the end of 2019, the Coronavirus disease 2019 (COVID-19) epidemic has swept the world (1). Countries around the world have adopted different protective measures, and the situation and evolution of the epidemic differ from country to country (2). To contain COVID-19 in China, local governments responded quickly. Under various powerful prevention and control measures, the epidemic spread in China was basically controlled in just over 2 months. The prevention and control was a huge success (3). The white paper “Fighting COVID-19: China in Action (4)” divides China's anti-COVID-19 process and measures into five stages, as shown in Figure 1. Under the ongoing prevention and control phase, the situation of COVID-19 in China is generally stable and controllable, with sporadic cases in some regions.

China has a vast territory and a large population. Except COVID-19, other major infectious diseases are among the top in the world. For example, there are more than 100 million hepatitis B and C virus infected people in China (5); the number of new cases of tuberculosis ranks the third in the world (6), which seriously affects public health, social and economic development and even national security. The spread of other infectious diseases cannot be ignored in the context of COVID-19. Studies have shown that these NPIs for COVID-19 may also reduce the incidence of respiratory infectious diseases such as influenza (7), as well as the number of other infectious diseases reported. Similar reports have been reported in the United States (8) and Germany (9). However, the long-term or short-term benefits of COVID-19 preventive and control interventions for other infectious diseases in China are still unclear, and there are still no studies based on national annual data.

Understanding the impact of the COVID-19 pandemic and related prevention and control measures on the incidence of other infectious diseases is of great significance for China's public health and infectious disease supervision. Therefore, this study collected the reported incidence data of all notifiable infectious diseases in China before and after the COVID-19 epidemic, and discussed the incidence trends of other notifiable infectious diseases under the epidemic prevention and control measures, so as to provide reference basis for further infectious disease surveillance, prevention and control work.

## Materials and Methods

### Data Collection

In this study, the incidence data of notifiable infectious diseases were obtained from the official website of the National Health Commission of the People's Republic of China (http://www.nhc.gov.cn/). The notifiable infectious diseases refer to the infectious diseases reported in accordance with the law stipulated in the “Law of the People's Republic of China on prevention and control of infectious diseases” (10), including a total of 40 infectious diseases in categories A, B, and C. In 2004, China established an infectious disease network reporting system, which realized the case, real-time, online reporting. This system covers in towns and townships and above all the medical and health institutions, by clinical doctors to complete fill in the diagnosis of infectious diseases and infectious diseases reporting card, and then be transcribed and reported by a specialist from the Public Health Department or the Preventive Health Department. Statistics on the incidence and death of notifiable infectious diseases will be published on the website of the National Health Commission every month. The population data were obtained from China Statistical Yearbook 2020 (11). There are no ethical issues involved in this study, the data is publicly available and accessible in accordance with the law. We obtained the monthly reported incidence data of all notifiable infectious diseases in 2019 and 2020, and the incidence of influenza and tuberculosis monthly reported in 2015–2020.

### Statistical Analysis

Excel 2016 was used for data summary processing, Graphpad Prism 9.0 was used for chart drawing, and SPSS 26.0 was used for data analysis. Using descriptive epidemiological survey method, the incidence (per 100,000) as the number of reported cases divided by the population size. The incidence differences of all notifiable infectious diseases in 2019 and 2020 were compared. The monthly reported data with normal distribution using the *t*-test, otherwise using the rank-sum test, and the annual total incidence and monthly increase rates and 95%CI were calculated. According to the transmission route, the notifiable infectious diseases were divided into respiratory infectious diseases, intestinal infectious diseases, blood and sexually transmitted diseases, natural foci and vector borne diseases, and several common ones in China were selected for specific analysis. Chi-squared test for trend was used to describe and judge the trend of diseases. The threshold for statistical significance was set at *P* < 0.05.

## Results

### Overview of the Incidence of Notifiable Infectious Diseases in China in 2020

In addition to COVID-19, a total of 6,366,176 cases of notifiable infectious diseases were reported nationwide in 2020, the total incidence rate was 454.71 cases/100,000. The highest incidence infectious disease was influenza (87.63 cases/100,000), followed by hepatitis B (81.36 cases/100,000), and then other infectious diarrhea (76.33 cases/100,000). The three diseases accounted for 53.95% of all the diseases. Compared with the same period in 2019, except the brucellosis, leptospirosis and kala-azar, other notifiable infectious diseases in 2020 decreased to varying degrees, with a year-on-year decrease of 41.38% in total, and category B and category C infectious diseases decreased by 14.84 and 54.98%, respectively, with statistical significance (*P* < 0.01). Dengue fever, rubella, and whooping cough experienced the greatest decrease in incidence. Influenza, hand-foot-mouth disease (HFMD) and other infectious diarrhea diseases had the largest decrease in reported cases (see attached Table 1 for details).

**Table 1:** Incidence of notifiable infectious diseases nationwide in 2019 and 2020.

**Category**	**Number of reported cases**	**Number of reported cases**	**Increase cases**	**Incidence rate (/ 100,000)**	**Incidence rate (/ 100,000)**	**Increase rate (95% CI)**	***t/z*-value**	***P***
	**2019**	**2020**		**2019**	**2020**			
Category A infectious diseases	21	15	/	/	/	/	/	/
Plague	5	4	/	/	/	/	/	/
Cholera	16	11	/	/	/	/	/	/
Category B infectious diseases (Except COVID−19)	3,679,089	3,133,258	−545,831	263.66	223.80	−14.84% (−14.88 to −14.80)	−3.349^*^	0.001
SARS	0	0	/	/	/	/	/	/
AIDS	72,630	63,154	−9,476	5.21	4.51	−13.05% (−13.30 to −12.81)	−1.559^*^	0.119
HAV	20,005	15,481	−4,524	1.43	1.11	−22.61% (−23.19 to −22.04)	3.901	0.001
HBV	1,247,092	1,139,133	−107,959	89.37	81.36	−8.66 % (−8.71 to −8.61)	−1.963^*^	0.0496
HCV	260,704	229,204	−31,500	18.68	16.37	−12.08% (−12.21 to −11.96)	−2.512^*^	0.012
HDV	411	217	−194	0.03	0.02	−47.20% (−52.03 to −42.42)	6.522	0.000
HEV	29,126	19,709	−9,417	2.09	1.41	−32.33% (−32.87 to −31.80)	−4.157^*^	0.000
Unclassified hepatitis	14,411	9,382	−5,029	1.03	0.67	−34.90% (−35.68 to −34.13)	−4.157^*^	0.000
Poliomyelitis	0	0	/	/	/	/	/	/
Human infection with highly pathogenic avian influenza	0	0	/	/	/	/	/	/
Measles	3,573	1,234	−2,339	0.26	0.09	−65.46% (−67.00 to −63.89)	−3.985^*^	0.000
Epidemic hemorrhagic fever	10,117	8,546	−1,571	0.73	0.61	−15.53% (−16.25 to −14.84)	−1.501^*^	0.133
Rabies	312	212	−100	0.02	0.02	−32.05% (−37.42 to −27.12)	3.334	0.003
Japanese encephalitis	463	312	−151	0.03	0.02	−32.61% (−37.01 to −28.50)	−0.665^*^	0.506
Dengue fever	22,317	802	−21,515	1.60	0.06	−96.41% (−96.65 to −96.16)	−3.465^*^	0.001
Anthrax	320	232	−88	0.02	0.02	−27.50% (−32.64 to −22.90)	−1.070^*^	0.285
Bacterial and amoebic dysentery	81,781	58,299	−23,482	5.86	4.16	−28.71% (−29.02 to −28.40)	1.900	0.071
Tuberculosis	1,034,760	876,576	−158,184	74.16	62.61	−15.29% (−15.36 to −15.22)	2.827	0.010
Typhoid and paratyphoid	9,787	7,321	−2,466	0.70	0.52	−25.20% (−26.07 to −24.35)	2.497	0.021
Epidemic cerebrospinal meningitis	124	57	/	/	/	/	/	/
Whooping cough	30,727	4,994	−25,733	2.20	0.36	−83.75% (−84.16 to −83.33)	−4.157^*^	0.000
Diphtheria	0	2	/	/	/	/	/	/
Neonatal tetanus	67	36	/	/	/	/	/	/
Scarlet fever	83,028	17,206	−65,822	5.95	1.23	−79.28% (−79.55 to −79.00)	−3.695^*^	0.000
Brucellosis	46,700	50,115	3,415	3.35	3.58	7.31% (7.08 to 7.55)	−0.486	0.632
Gonorrhea	120,146	106,592	−13,554	8.61	7.61	−11.28% (−11.46 to −11.10)	−0.346^*^	0.729
Syphilis	587,402	522,920	−64,482	42.10	37.35	−10.98% (−11.06 to −10.90)	−2.656^*^	0.008
Leptospirosis	226	315	89	0.02	0.02	39.38% (33.24 to 45.88)	−0.145^*^	0.885
Schistosomiasis	224	67	/	/	/	/	/	/
Malaria	2,635	1,140	−1,495	0.19	0.08	−56.74% (−58.62 to −54.84)	−3.465^*^	0.001
Human infection with H7N9 bird flu	1	0	/	/	/	/	/	/
COVID−19	/	86,746	/	/	/	/	/	/
Category C infectious diseases	7,181,455	3,232,903	−3,948,552	514.66	230.91	−54.98% (−55.02 to −54.94)	−3.406^*^	0.001
Influenza	3,507,306	1,226,804	−2,280,502	251.35	87.63	−65.02% (−65.07 to −64.97)	−3.349^*^	0.001
Mumps	303,105	130,911	−17,2194	21.72	9.35	−56.81% (−56.99 to −56.63)	5.968	0.000
Rubella	34,151	2,839	−31,312	2.45	0.20	−91.69% (−91.98 to −91.39)	−3.811^*^	0.000
Acute hemorrhagic conjunctivitis	41,701	28,633	−13,068	2.99	2.05	−31.34% (−31.79 to −30.90)	3.777	0.002
Leprosy	531	449	−82	0.04	0.03	−15.44% (−18.76 to −12.62)	1.328	0.198
Typhus	1,201	1,199	−2	0.09	0.09	−0.17% (−0.61 to −0.05)	0.008	0.993
Kala–azar	178	231	53	0.01	0.02	29.78% (23.55 to 36.87)	−1.876	0.074
Echinococcosis	4,850	3,739	−1,111	0.35	0.27	−22.91% (−24.11 to −21.75)	2.836	0.010
Filariasis	0	0	/	/	/	/	/	/
Other infectious diarrhoeal diseases	1,344,396	106,8650	−275,746	96.35	76.33	−20.51% (−20.58 to −20.44)	2.563	0.018
HFMD	1,944,036	769,448	−1,174,588	139.32	54.96	−60.42% (−60.49 to −60.35)	−2.367^*^	0.018
Total (excluding COVID−19)	10,860,565	6,366,176	−449,4389	778.32	454.71	−41.38% (−41.41 to −41.35)	−3.522^*^	0.000

No comparison was made for diseases with reported numbers of ≤ 100 cases. The total number of reported cases is the sum of the monthly number of reported cases; ^*^is rank sum test, others are t-test (HFMD, Hand–Foot–Mouth Disease; HAV, Hepatitis A; HBV, Hepatitis B; HCV, Hepatitis C, HDV, Hepatitis D; HEV, Hepatitis E; SARS, Severe Acute Respiratory Syndrome; AIDS, Acquired Immune Deficiency Syndrome).

### Incidence Trends of Common Notifiable Infectious Diseases

**Figure 2:**
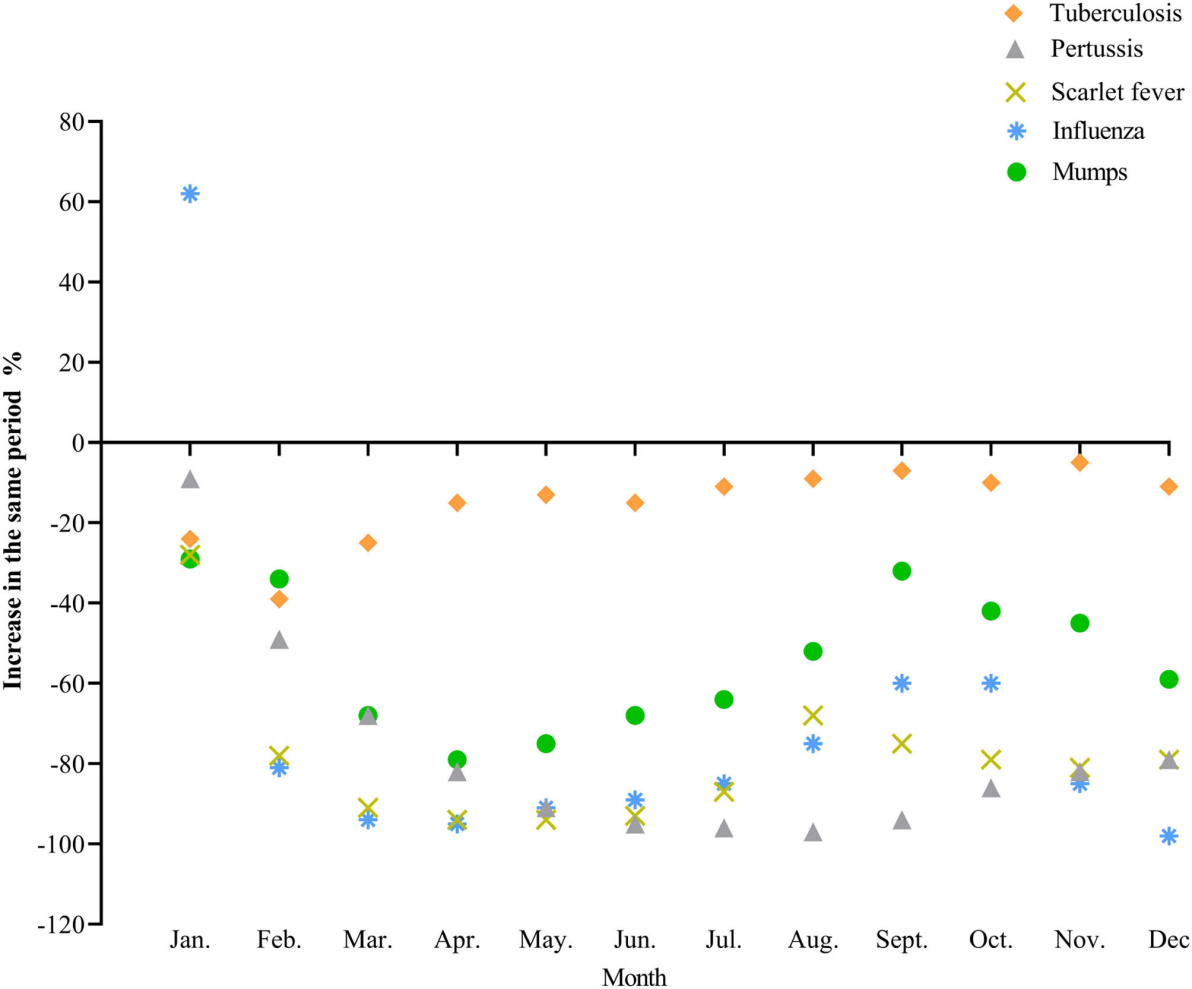
The incidence trend of common respiratory diseases in 2020.

We found that there are differences in the incidence of infectious diseases in different transmission routes. Compared with the same period in 2019, the prevalence of respiratory infectious diseases in China has changed most obviously in 2020. As shown in Figure 2, except for the increase in influenza in January, the monthly reported incidence of other common respiratory infectious diseases was seen to decrease significantly, with the largest decline from April to May. Although the overall growth of tuberculosis was negative, the decline was not significant compared with that of other respiratory infectious diseases.

**Figure 3:**
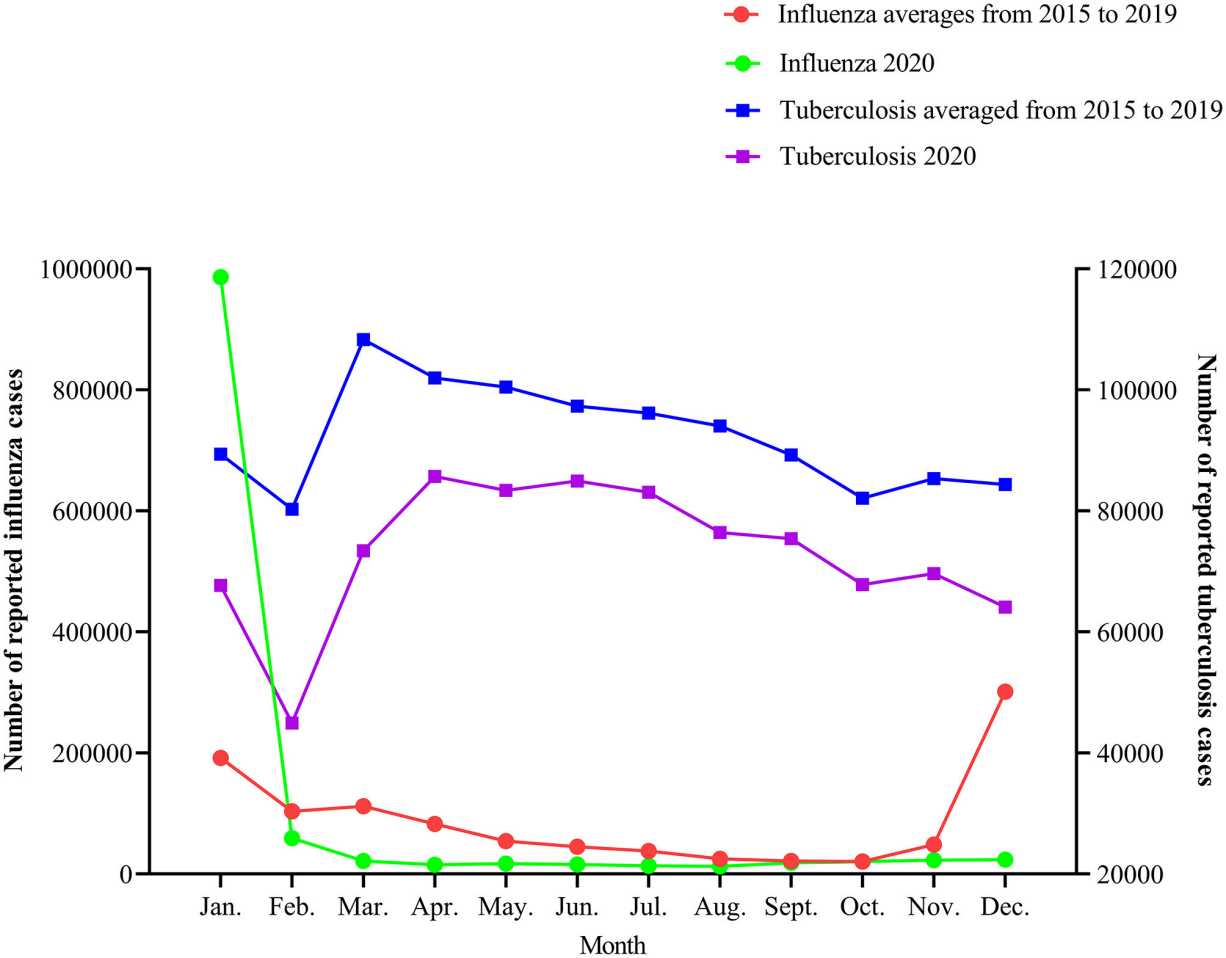
Influenza and tuberculosis epidemics 2015–2020.

As we can see in Figure 3, In 2020, the incidence of influenza peaked in early January, decreased sharply in February, reached the lowest level in April, and remained at a relatively low level; The monthly variation curve of tuberculosis epidemic is similar to that in previous years, the number of cases decreased significantly in February and gradually recovered from March to April. The incidence trend of influenza and tuberculosis in 2020 were lower than the average from the last 5 years (*P* for trend <0.000).

**Figure 4:**
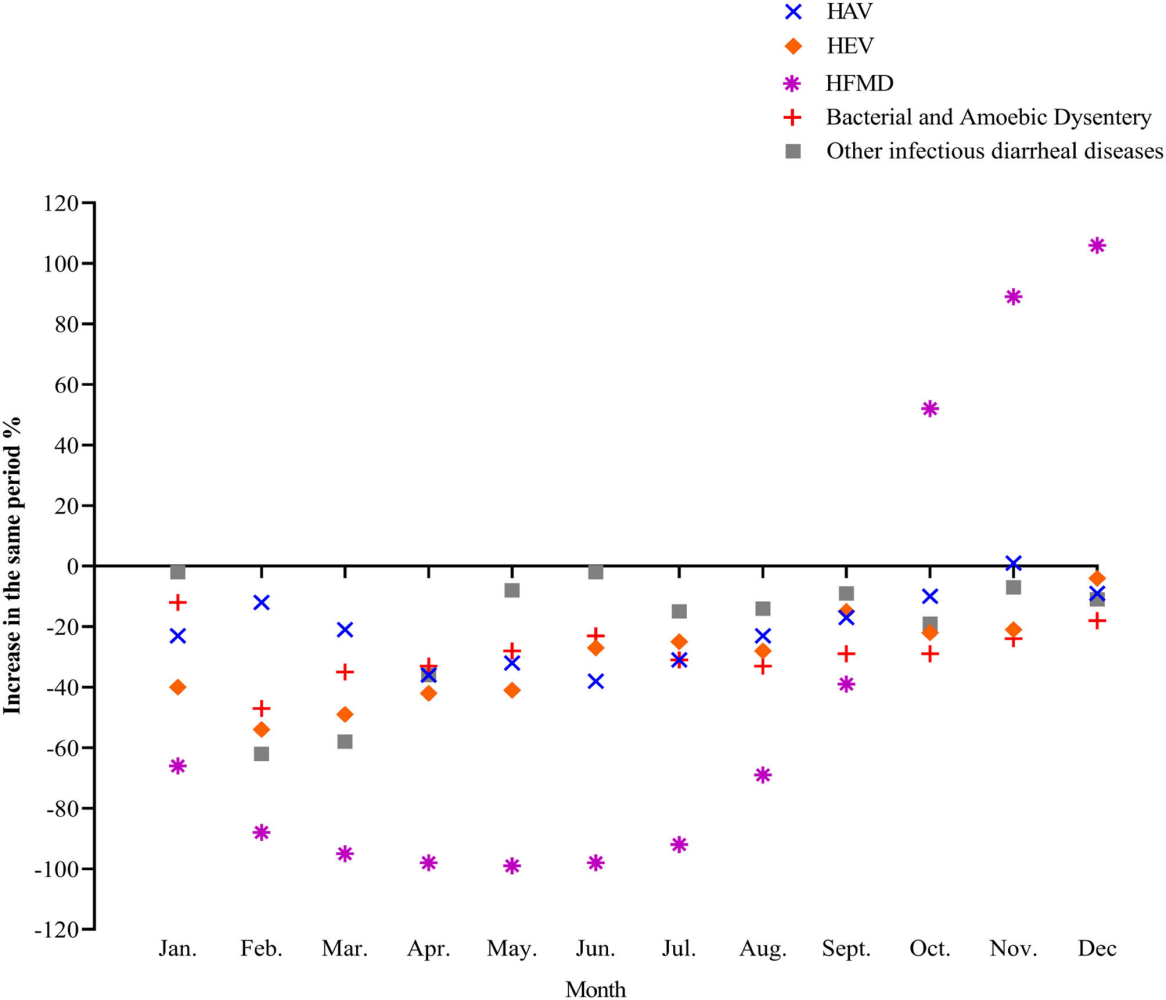
The incidence trend of common intestinal infectious diseases in 2020.

In 2020, the incidence of four common intestinal infectious diseases, hepatitis A, hepatitis E, bacterial amoebic dysentery, and other infectious diarrhea diseases, decreased substantially. Although the decrease was not significant compared with several common respiratory infectious diseases, the incidence was still lower than that of the same period in 2019. The incidence of HFMD fluctuated the most, decreasing first and then increasing. The number of reported monthly incidences from March to June dropped by more than 95% year-on-year, and from October to December it was higher than the same period in 2019 (Figure 4).

**Figure 5:**
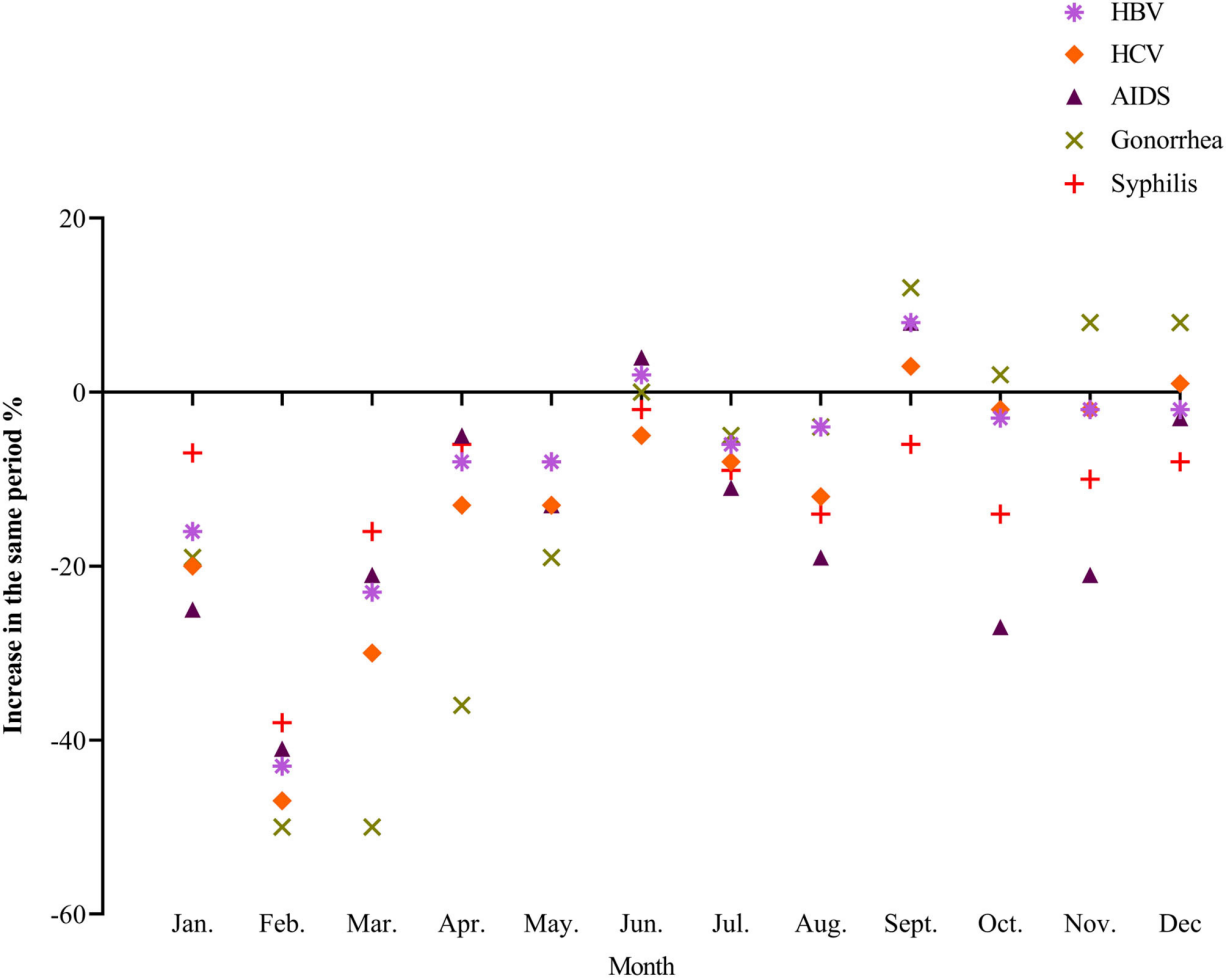
The incidence trend of common blood-origin and sexual transmitted diseases in 2020.

Compared with 2019, the monthly incidence of several common blood-borne and sexually transmitted diseases in China in 2020 was still on a downward trend for the most part, with a relatively obvious decline in February 2020 and then a fluctuating recovery. Since June, the incidence of AIDS, gonorrhea and syphilis was even higher than that of the same period in 2019 (Figure 5).

**Figure 6:**
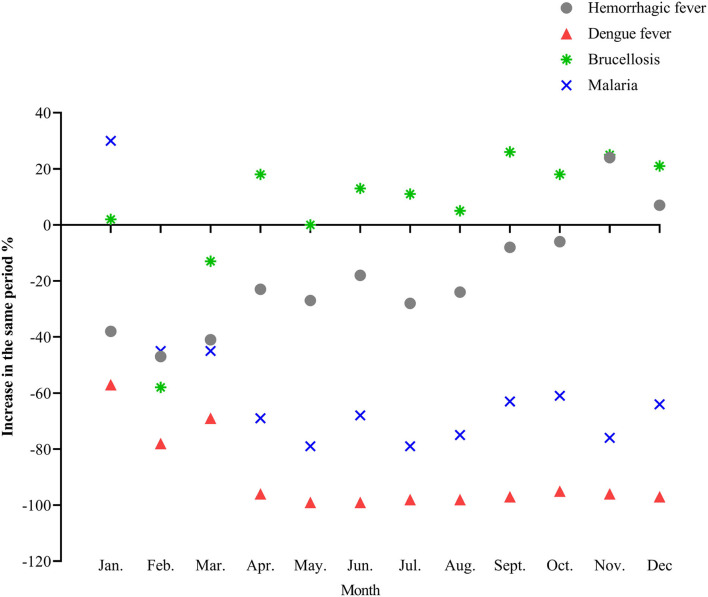
The incidence trend of natural foci and insect-borne diseases in 2020.

Compared with the same period in 2019, the incidence rate of dengue fever and malaria decreased significantly in 2020. Dengue fever cases have dropped by more than 95% since April. The overall incidence of brucellosis was higher than that in 2019 (Figure 6). The incidence data of all the above diseases are detailed in the Supplementary Materials.

## Discussion

In generally, compared with 2019 the total incidence of categories A, B, and C notifiable infectious diseases in China in 2020 showed a decreasing trend (*P* < 0.05), suggesting that the COVID-19 prevention and control measures may have a good prevention effect on most other infectious diseases, and the risk of spread of infectious diseases is reduced.

Under COVID-19 prevention and control measures, the prevalence of respiratory infectious diseases in China has changed most obviously, and the decline incidence is closely related to the prevention and control measures at different stages. At the end of January 2020, China launched an emergency response to the epidemic and fully implemented prevention and control measures. Personal protective measures such as wearing masks, hand hygiene and social distancing have effectively prevented the spread of respiratory infections through droplets and aerosols. Measures such as school closures, home quarantine, transportation restrictions, and public place closures have significantly reduced population movement, reducing the probability of exposure to the virus and the risk of cross-infection among susceptible people (12, 13). The survey showed that during the Spring Festival travel rush in 2020, the passenger flow of China's railways, highways, waterways, and aviation dropped by more than 50% year-on-year compared with 2018 and 2019 (14). At the same time, the closure of small and medium-sized medical institutions, public panic and heightened awareness of risk prevention may lead to a gradual decrease in the number of medical treatments, some mild patients were covered up (15), and the reporting rate of infectious diseases decreased. Every year, the period from March to May is the peak of pertussis, measles, and other respiratory infectious diseases. However, in 2020, this peak did not appear and the number of cases decreased, or even a trough period. At the end of April, the epidemic entered the stage of normal prevention and control. With the steady progress of the resumption of work, production, schools, and the resumption of normal production and living order, the incidence of common respiratory infectious diseases began to slowly and modestly recover, with the decline decreasing, but still lower than that of the same period in 2019.

The results of this study showed that the prevalence trends of influenza and tuberculosis in 2020 were significantly lower than the average of the previous 5 years (*P* < 0.05). Influenza is the most common infectious disease in China. In recent 10 years, the incidence of seasonal influenza in China has been increasing year by year. In January 2020, the winter peak of influenza in China coincided with the outbreak of COVID-19. The large number of influenza reported in January may be the result of changes in its natural epidemic trend, while the sharp drop in February may be due to COVID-19 containment measures limiting the spread of influenza. Public data from the National Influenza Center of China shows that from the 5th week of 2020 (January 27, 2020 to February 02, 2020), the total testing number and the positive rate of influenza decreased significantly. In the 7th week (from February 10, 2020 to February 16, 2020) the positive rate of influenza was significantly lower than that in 2016–2019 (16). The high-incidence period of influenza in China in 2020 ended ahead of schedule. We can see that even in the second half of the year, the COVID-19 epidemic has entered the normalized prevention and control stage, the reporting and testing of influenza have resumed and is even stricter than previously required, the incidence of influenza is still lower than that of the same period in the previous 5 years, indicating that the incidence of influenza has indeed declined. Although the response time and epidemic prevention measures of various countries and regions to COVID-19 were different, as well as slightly different epidemic trends of influenza, studies in Taiwan, Hong Kong, the United States, South Korea, and Europe have also obtained similar results. Overall, the seasonal duration of influenza was shortened under COVID-19 prevention and control, and the influenza positive rate declined rapidly within 7–12 weeks of 2020 (17–20).

Pulmonary tuberculosis has a delitescence onset and has no specificity the incubation period. The period is generally long, and the diseases can occur with in a few months to 1 year (or even over a longer time). People are generally susceptible to the disease. Compared with COVID-19, although it is also a respiratory disease, tuberculosis is mainly concentrated in close contacts in small areas, so the management of infection source is the key to controlling the spread of tuberculosis. As suggested in this study, a series of COVID-19 control measures might reduce the community transmission of tuberculosis. However, China has a large base of tuberculosis patients, high drug resistance rate, unsatisfactory therapeutic effect, and low cure rate (21). During the outbreak period, the closure of many medical institutions and emergency clinics, the suspension of admission of tuberculosis patients in some designated tuberculosis hospitals, the decrease in the number of tuberculosis screening patients, and the delay or interruption of treatment of tuberculosis patients may increase the risk of potential tuberculosis infection and the probability of chronic transmission. Therefore, the prevention and control effect of the COVID-19 epidemic on tuberculosis and the epidemic trend still require more detailed long-term evaluations (22).

The results of this study showed that the incidence of common intestinal infectious diseases in China in 2020 were lower than that in the same period in 2019, among which there were statistically significant differences in the incidence of hepatitis A, hepatitis E, HFMD, and other infectious diarrhea (*P* < 0.05). It shows that the NPIs for COVID-19, such as continuous monitoring of epidemic information, standardized operation of food and entertainment, national conscious epidemic prevention mechanism, related knowledge and health education, may played a certain role in the prevention and control of intestinal infectious diseases. The incidence of HFMD fluctuates greatly, the sharp drop in the number of cases in the first half of the year could be related to protective measures such as post-ponement of school, home isolation and travel restrictions. At the same time, these measures can also conducive to preventing other children's susceptible diseases such as mumps (23). HFMD frequently occurs in autumn and winter, and the school starts in the second half of the year, the population was dense. Infants and children have low immunity, lack of ability to develop healthy behaviors, and poor implementation of hand hygiene and other protective measures, which can easily causes the spread of diseases and increase the incidence (24).

During the COVID-19 outbreak in February 2020, the incidence of blood-borne, and sexually transmitted diseases decreased rapidly. Some strict epidemic control measures like prohibiting gatherings and closing hotels, KTV, and other dangerous places resulted in a decrease in people's social activities, and a significant decrease in high-risk groups such as whoring, men who have sex with men, migrant workers, drug users, and sexually transmitted diseases patients (25), which could directly affect the spread of related blood sources and sexually transmitted diseases. When entering the stage of normal prevention and control, the incidence of some blood-borne and sexually transmitted diseases, such as AIDS, syphilis, and gonorrhea, was even higher than the same period in 2019. At the same time, there was little change in the incidence of hepatitis B in the past 2 years (*P* > 0.05), suggesting that the incidence of blood-borne and sexually transmitted diseases under the normal epidemic prevention and control has been impacted to some extent, although the change in its epidemic trend is not obvious (26, 27). In the future, it will still be necessary to strengthen the monitoring of key high-risk groups, restrict drug abuse, dangerous sex, and other high-risk activities, strengthen publicity and education, improve the public's self-protection awareness, and further search for more effective prevention and control strategies.

The incidence of natural foci and insect-borne infectious diseases varies greatly with the different stages. In 2020, the incidence of dengue fever decreased significantly compared with the same period in 2019, and the monthly decrease since April was more than 95%, which may be related to the global outbreak of dengue fever in 2019 and the surge of dengue epidemic cases in many places in China (28). China has strengthened control over people living abroad following the COVID-19 outbreak, which also limited the spread of imported diseases such as dengue fever and malaria. At the same time, the country forbids the consumption of live wild animals, and people's awareness of protection is enhanced, which also reduces the possibility of cross-border transmission of infectious diseases to a certain extent (29). The year-on-year increase in the incidence of brucellosis was related to the “Lanzhou Event” in China in 2020, which led to the outbreak of brucellosis, the increase in detection quantity, and the increase in the number of reported cases (30). Other natural foci and insect-borne diseases have a relatively small number of people, are diverse and complex, and have a certain degree of occasionality. The current NPIs for COVID-19 have no clear impact on natural foci and insect-borne infectious diseases. In the future, we should pay more attention to the incidence trend of these diseases, early monitoring and early warning, adopt comprehensive prevention and control strategies, and take measures according to local conditions and time conditions to prevent and control its occurrence and development.

Scientific and reasonable epidemic prevention measures not only seem to reduce the incidence of infectious diseases and maintain public health, but also promote the recovery and development of social economy to a certain extent. In the early days of the COVID-19 outbreak, social production was disrupted, and cities such as Wuhan were temporarily shut down. However, China's rapid response strategy has effectively controlled the spread of COVID-19 and reversed the trend of economic decline. In the post-epidemic period, China paid equal attention to the resumption of work and production and epidemic prevention and control, effectively balancing epidemic control and economic recovery (31, 32). Up to now, China has maintained a very low level of COVID-19 transmission. At the same time, China became the only major economy in the world with positive growth in 2020. It is suggested that taking such prevention and control measures to control infectious diseases is of great value for social and economic development. These experiences are worth learning from all over the world.

## Limitation

There are some limitations in this study. First, the epidemic of infectious disease is affected by multiple factors, it is difficult to obtain data on the interaction between vaccination and population immunity, seasonal and climate change, travel and human mobility, and virus variation. Second, the case data in this study came from passive monitoring reports of various places, which may lead to data bias due to missing reports, and the actual incidence may be underestimated. At present, it cannot be concluded that there is a direct causal relationship between the prevention and control measures of COVID-19 at different stages and the decrease of the incidence of related diseases. We cannot fully explain this by the monthly reported incidence data alone, and a decrease in the reported data, while revealing some issues, does not necessarily mean changes in the true prevalence and incidence. We can only interpret and analyze the possible influencing factors currently available. However, these strict non-pharmaceutical prevention and control measures may be a useful supplement to the prevention and control of related respiratory and intestinal infectious diseases. In the next steps, we will continue to strengthen surveillance and comprehensively assess the long-term impact of COVID-19 prevention and control measures on other notifiable infectious diseases.

## Conclusion

In conclusion, under the COVID-19 prevention and control measures, the changes in incidence of notifiable infectious diseases in China generally showed a downward trend, among which, respiratory infectious diseases showed the most obvious decline. Public health interventions such as frequent hand washing, wearing masks, cough etiquette, and social distance provide certain references for the prevention and control of other infectious diseases in the future.

## Data Availability Statement

The original contributions presented in the study are included in the article/Supplementary Material, further inquiries can be directed to the corresponding authors.

## Author Contributions

BC, ZL, and PZ: design the study. MW, MX, LP, and HL: contribute to data acquisition. MW and LP: contribute to data analysis. BC and MW: write the manuscript. BC, MW, XH, and PZ: revise the manuscript. All authors contributed to manuscript revision, read, and approved the submitted version.

## Funding

This work was supported by the Science Popularization Project of Innovative Province Construction in Hunan (grant no: 2021ZK4198) and the Central South University COVID-19 Prevention and Control Emergency Project (grant no: 160260003).

## Conflict of Interest

The authors declare that the research was conducted in the absence of any commercial or financial relationships that could be construed as a potential conflict of interest.

## Publisher's Note

All claims expressed in this article are solely those of the authors and do not necessarily represent those of their affiliated organizations, or those of the publisher, the editors and the reviewers. Any product that may be evaluated in this article, or claim that may be made by its manufacturer, is not guaranteed or endorsed by the publisher.
