# Supplementary material for: Changes in Incidence of Notifiable Infectious Diseases in China Under the Prevention and Control Measures of COVID-19
Source: Front Public Health. 2021 Oct 15;9:728768. doi: 10.3389/fpubh.2021.728768 (PMC8553983; doi:10.3389/fpubh.2021.728768)
Supplement: Supplementary file 1 [file Data_Sheet_1.PDF]

Supplementary table 1 The incidence of common respiratory infectious diseases in 2020

Month	Number of cases of tuberculosis (cases)			Number of cases of pertussis (cases)			Number of cases of scarlet fever (cases)			Number of cases of influenza (cases)			Number of cases of mumps (cases)		
	2019	2020	Increase rate% (95% CI)	2019	2020	Increase rate% (95% CI)	2019	2020	Increase rate% (95% CI)	2019	2020	Increase rate% (95% CI)	2019	2020	Increase rate% (95% CI)
Jan.	88597	67682	-23.61(-23.89 to -23.33)	1260	1141	-9.44 (-11.18 to -7.95)	8838	6352	-28.13(-29.08 to -27.20)	608511	986543	62.12(62.00 to 62.24)	24075	17159	-28.73(-29.30 to -28.16)
Feb.	73096	44933	-38.53(-38.88 to -38.18)	1444	738	-48.89(-51.47 to -46.32)	2591	580	-77.61(-79.17 to -75.96)	307892	59154	-80.79(-80.93 to -80.65)	12169	8018	-34.11(-34.96 to -33.27)
Mar.	97866	73427	-24.97 (-25.24 to -24.70)	2735	874	-68.04(-69.76 to -66.27)	5120	444	-91.33(-92.07 to -90.53)	358757	21696	-93.95(-94.03 to -93.87)	18827	6028	-67.98(-68.64 to -67.31)
Apr.	101191	85684	-15.32(-15.54 to -15.10)	2689	479	-82.19(-83.59 to -80.70)	6964	442	-93.65(-94.20 to -93.05)	299939	15396	-94.87(-94.95 to -94.79)	28735	6169	-78.53(-79.00 to -78.05)
May.	96106	83385	-13.24(-13.46 to -13.03)	3129	277	-91.15(-92.10 to -90.10)	9087	562	-93.82 (-94.30 to -93.31)	197729	16974	-91.42(-91.54 to -91.30)	36669	9273	-74.71(-75.15 to -74.26)
Jun.	99555	84952	-14.67(-14.89 to -14.45)	3128	159	-94.92(-95.61 to -94.06)	10052	677	-93.27(-93.74 to -92.76)	141202	15640	-88.92(-89.08 to -88.75)	37913	11995	-68.36(-68.83 to -67.89)
Jul.	93318	83101	-10.95(-11.15 to -10.75)	3635	131	-96.40(-96.96 to -95.74)	5864	789	-86.55(-87.40 to -85.65)	88911	13406	-84.92(-85.15 to -84.68)	29427	10583	-64.04(-64.59 to -63.49)
Aug.	84304	76423	-9.35(-9.55 to -9.16)	4388	142	-96.76(-97.24 to -96.19)	2396	763	-68.16(-69.99 to -66.27)	51676	12833	-75.17(-75.54 to -74.79)	18690	9008	-51.80(-52.52 to -51.08)
Sept.	80973	75409	-6.87(-7.05 to -6.70)	3319	201	-93.94(-94.70 to -93.08)	3473	877	-74.75(-76.17 to -73.28)	46048	18432	-59.97(-60.42 to -59.52)	19377	13212	-31.82(-32.48 to -31.17)
Oct.	75123	67843	-9.69(-9.90 to -9.48)	1933	267	-86.19(-87.66 to -84.58)	5375	1102	-79.50(-80.56 to -78.40)	50665	20401	-59.73(-60.16 to -59.30)	21938	12818	-41.57(-42.22 to -40.92)
Nov.	73000	69640	-4.60(-4.75 to -4.45)	1653	291	-82.40(-84.16 to -80.49)	10215	1925	-81.16(-81.91 to -80.39)	156205	22783	-85.41(-85.58 to -85.23)	27704	15211	-45.09(-45.68 to -44.50)
Dec.	71631	64097	-10.52(-10.75 to -10.30)	1414	294	-79.21(-81.24 to -77.02)	13053	2693	-79.37(-80.06 to -78.67)	1199771	23546	-98.04(-98.06 to -98.02)	27581	11437	-58.53(-59.11 to -57.95)

Supplementary table 2 The incidence of common intestinal infectious diseases in 2020

Month	Number of cases of HAV (cases)			Number of cases of HEV (cases)			Number of cases of bacterial and amoebic dysentery(cases)			Number of cases of HFMD(cases)			Number of cases of other infectious diarrheal diseases(cases)		
	2019	2020	Increase rate% (95% CI)	2019	2020	Increase rate% (95% CI)	2019	2020	Increase rate% (95% CI)	2019	2020	Increase rate% (95% CI)	2019	2020	Increase rate% (95% CI)
Jan.	1622	1251	-22.87(-24.98 to-20.89)	2695	1626	-39.67(-41.53to-37.84)	3774	3309	-12.32(-13.41to-11.31)	83676	28355	-66.11(-66.43to-65.79)	124502	121473	-2.43(-2.52to-2.35)
Feb.	1465	1295	-11.60(-13.34 to-10.06)	2280	1045	-54.17(-56.21to-52.12)	3628	1912	-47.30(-48.93to-45.68)	28196	3263	-88.43(-88.80to-88.05)	120881	45510	-62.35(-62.62to-62.08)
Mar.	1938	1529	-21.10(-22.97 to-19.34)	3204	1641	-48.78(-50.51to-47.05)	4423	2892	-34.61(-36.02to-33.22)	53938	2869	-94.68(-94.87to-94.49)	116329	48491	-58.32(-58.60to-58.04)
Apr.	1864	1187	-36.32(-38.53 to-34.17)	3010	1732	-42.46(-44.23to-40.7)	5556	3695	-33.50(-34.75to-32.27)	155696	3001	-98.07(-98.14to-98.00)	91421	58544	-35.96(-36.27to-35.65)
May.	1615	1100	-31.89(-34.20 to-29.66)	2677	1585	-40.79(-42.66to-38.94)	7849	5664	-27.84(-28.84to-26.86)	268630	3919	-98.54(-98.58to-98.49)	94564	87151	-7.84(-8.01to-7.67)
Jun.	2027	1249	-38.38(-40.52 to-36.29)	2346	1708	-27.20(-29.04to-25.44)	10146	7775	-23.37(-24.2to-22.56)	337393	6260	-98.14(-98.19to-98.09)	116800	114085	-2.32(-2.41to-2.23)
Jul.	2203	1514	-31.28(-33.25 to-29.38)	2462	1839	-25.30(-27.05to-23.62)	11468	7943	-30.74(-31.59to-29.90)	339521	26778	-92.11(-92.20to-92.02)	136998	116425	-15.02(-15.21to-14.83)
Aug.	1809	1401	-22.55(-24.53 to-20.68)	2436	1759	-27.79(-29.60to-26.05)	10871	7270	-33.12(-34.01to-32.24)	193230	59143	-69.39(-69.6to-69.18)	127630	109407	-14.28(-14.47to-14.09)
Sept.	1603	1335	-16.72(-18.63 to-14.97)	2123	1811	-14.70(-16.27to-13.26)	8709	6206	-28.74(-29.70to-27.80)	188477	115170	-38.89(-39.11to-38.67)	106258	97075	-8.64(-8.81to-8.47)
Oct.	1313	1181	-10.05(-11.79 to-8.54)	1943	1506	-22.49(-24.4to-20.69)	6707	4793	-28.54(-29.63to-27.47)	131252	199875	52.28(52.01to52.55)	97658	79196	-18.90(-19.15to-18.66)
Nov.	1230	1243	1.05(0.62 to1.80)	2062	1639	-20.51(-22.31to-18.82)	4680	3568	-23.76(-25.00to-22.56)	100161	189017	88.71(88.51to88.90)	89252	83209	-6.77(-6.94to-6.61)
Dec.	1316	1196	-9.12(-10.80 to-7.65)	1888	1818	-3.71(-4.66 to-2.95)	3970	3272	-17.58(-18.8to-16.43)	63866	131798	106.37(106.17to106.58)	122103	108084	-11.48(-11.66to-11.30)

Supplementary table 3 The incidence of common blood origin and sexual transmitted diseases in 2020

Month	Number of cases of HBV (cases)			Number of cases of HCV (cases)			Number of cases of AIDS(cases)			Number of cases of gonorrhea(cases)			Number of cases of syphilis(cases)		
	2019	2020	Increase rate% (95% CI)	2019	2020	Increase rate% (95% CI)	2019	2020	Increase rate% (95% CI)	2019	2020	Increase rate% (95% CI)	2019	2020	Increase rate% (95% CI)
Jan.	107754	91026	-15.52(-15.74 to -15.31)	21512	17287	-19.64(-20.18 to -19.11)	3688	2759	-25.19(-26.62 to -23.82)	10149	8254	-18.67(-19.44 to -17.92)	42795	39671	-7.30(-7.55 to -7.06)
Feb.	90985	51506	-43.39(-43.71 to -43.07)	17214	9068	-47.32(-48.07 to -46.57)	3587	2133	-40.54 (-42.16 to -38.94)	7059	3524	-50.08(-51.25 to-48.91)	34695	21448	-38.18(-38.69 to -37.67)
Mar.	113941	88150	-22.64(-22.88 to -22.40)	24053	16718	-30.50(-31.08 to -29.92)	6086	4808	-21.00(-22.04 to -20.00)	9413	4661	-50.48(-51.49 to -49.47)	49274	41154	-16.48(-16.81 to -16.16)
Apr.	110266	101262	-8.17 (-8.33 to -8.01)	23218	20179	-13.09(-13.53 to -12.66)	6277	5960	-5.05(-5.62 to -4.54)	9810	6267	-36.12(-37.08 to -35.18)	49861	46728	-6.28(-6.50 to -6.07)
May.	106431	97651	-8.25(-8.42 to -8.09)	22748	19821	-12.87(-13.31 to -12.44)	6291	5484	-12.83(-13.68 to -12.03)	10018	8104	-19.11(-19.89 to -18.35)	50895	46753	-8.14(-8.38 to -7.91)
Jun.	97362	99319	2.01 (1.92 to 2.10)	21419	20367	-4.91(-5.21 to -4.63)	6642	6915	4.11(3.66 to 4.61)	9321	9292	-0.31(-0.45 to -0.22)	47675	46538	-2.38(-2.52 to -2.25)
Jul.	112454	106135	-5.62 (-5.76 to -5.49)	24393	22400	-8.17(-8.52 to -7.83)	6912	6124	-11.40 (-12.17 to -10.67)	11204	10621	-5.20(-5.63 to -4.80)	55367	50386	-9.00(-9.24 to -8.76)
Aug.	106985	102304	-4.38 (-4.50 to -4.26)	23334	20520	-12.06(-12.48 to -11.65)	6404	5166	-19.33 (-20.32 to -18.38)	11114	10724	-3.51(-3.87 to -3.18)	54217	46838	-13.61 (-13.90 to -13.32)
Sept.	97815	105377	7.73 (7.56 to 7.90)	20866	21538	3.22(2.99 to 3.47)	6435	6927	7.65(7.03 to 8.32)	10425	11643	11.68(11.08 to12.31)	51981	48965	-5.80 (-6.00 to -5.60)
Oct.	98774	95633	-3.18 (-3.29 to -3.07)	20438	20067	-1.82(-2.01 to -1.65)	6207	4546	-26.76(-27.88 to -25.67)	10382	10551	1.63(1.40 to 1.89)	51616	44438	-13.91(-14.21 to -13.61)
Nov.	102174	100561	-1.58 (-1.66 to -1.51)	21182	20801	-1.80 (-1.99 to -1.63)	7366	5824	-20.93 (-21.87 to -20.02)	10448	11260	7.77(7.27 to 8.30)	50439	45305	-10.18 (-10.45 to -9.92)
Dec.	102151	100209	-1.90 (-1.99 to -1.82)	20327	20438	0.55(0.46 to 0.66)	6735	6508	-3.37(-3.83 to -2.96)	10803	11691	8.22 (7.72 to 8.75)	48587	44696	-8.01(-8.25 to -7.77)

Supplementary table 4 The incidence of common natural foci and insect-borne diseases in 2020

Month	Number of cases of epidemic hemorrhagic fever (cases)			Number of cases of dengue fever (cases)			Number of cases of brucellosis (cases)			Number of cases of malaria (cases)		
	2019	2020	Increase rate %(95% CI)	2019	2020	Increase rate% (95% CI)	2019	2020	Increase rate% (95% CI)	2019	2020	Increase rate% (95% CI)
Jan.	1109	684	-38.32(-41.22 to -35.46)	165	71	-56.97(-64.28 to -49.34)	2390	2445	2.30(1.77 to 2.98)	268	348	29.85(24.51 to 35.58)
Feb.	705	374	-46.95(-50.64 to -43.22)	78	17	-78.21(-85.93 to -67.84)	2227	933	-58.11(-43.95 to -39.86)	230	126	-45.22(-51.68 to -38.71)
Mar.	738	433	-41.32(-44.92 to -37.76)	52	16	-69.23(-80.08 to -55.73)	4021	3508	-12.75(-13.83 to -11.76)	167	92	-44.91(-52.48 to -37.56)
Apr.	701	540	-22.97(-26.23 to -19.94)	142	6	-95.77(-98.05 to -91.08)	4559	5360	17.57(16.49 to 18.70)	175	54	-69.14(-75.51 to -61.95)
May.	939	686	-26.94(-29.87 to -24.15)	330	4	-98.79(-99.53 to -96.93)	5238	5264	0.50(0.34 to 0.73)	195	40	-79.49(-84.56 to -73.28)
Jun.	1007	826	-17.97(-20.46 to -15.6)	788	4	-99.49(-99.80 to -98.70)	5484	6193	12.93(12.07 to 13.84)	231	74	-67.97(-73.65 to -61.70)
Jul.	703	503	-28.45(-31.97 to -25.1)	1260	23	-98.17(-98.78 to -97.27)	5791	6437	11.16(10.37 to 12.00)	261	54	-79.31(-83.78 to -73.99)
Aug.	411	313	-23.84(-28.19 to -19.86)	3311	55	-98.34(-98.72 to -97.85)	4755	4972	4.56(3.99 to 5.19)	226	57	-74.78(-80.00 to -68.74)
Sept.	346	320	-7.51(-10.78 to -5.06)	8036	247	-96.93(-97.29 to -96.53)	3561	4492	26.14(24.72 to 27.61)	220	81	-63.18(-69.28 to -56.63)
Oct.	649	611	-5.86(-7.94 to -4.23)	6120	287	-95.31(-95.81 to -94.75)	2717	3206	18.00(16.60 to 19.49)	200	78	-61.00(-67.49 to -54.09)
Nov.	1445	1796	24.29(22.12 to 26.57)	1767	63	-96.43(-97.20 to -95.46)	2892	3611	24.86(23.32 to 26.47)	258	63	-75.58(-80.42 to -69.99)
Dec.	1364	1460	7.04(5.77 to 8.52)	268	9	-96.64(-98.22 to -93.74)	3065	3694	20.52(19.13 to 21.99)	204	73	-64.22(-70.48 to -57.44)

Supplementary table 5 Influenza and tuberculosis epidemics 2015~2020

Month	Number of cases of Influenza (cases)					Number of cases of tuberculosis (cases)				
	Averages from 2015 to 2019	Incidence in 2015 to 2019（/100,000）	2020	Incidence in 2020 （/100,000）	Increase rate% (95% CI)	Averaged from 2015 to 2019	Incidence in 2015 to 2019 （/100,000）	2020	Incidence in 2020 （/100,000）	Increase rate% (95% CI)
Jan.	191946	13.89	986543	70.46	413.97(412.37 to 415.40)	70445.27	6.47	67682	4.83	-3.92(-4.07 to -3.78)
Feb.	103342	7.48	59154	4.23	-42.76(-43.06 to -42.46)	65168.19	5.81	44933	3.21	-31.05(-31.41to -30.70)
Mar.	111811.80	8.09	21696	1.55	-80.60(-80.83 to -80.37)	85633.83	7.84	73427	5.24	-14.25(-14.49 to -14.02)
Apr.	82876.80	6.00	15396	1.10	-81.42(-81.68 to -81.15)	80371.26	7.38	85684	6.12	6.61 (6.44 to 6.78)
May.	54264	3.93	16974	1.21	-68.72(-69.11 to -68.33)	80045.58	7.27	83385	5.96	4.17 (4.03 to 4.31)
Jun.	44817.60	3.24	15640	1.12	-65.10(-65.54 to -64.66)	76831.49	7.04	84952	6.07	10.57 (9.75 to 10.79)
Jul.	37850	2.74	13406	0.96	-64.58(-65.06 to -64.10)	75570.99	6.96	83101	5.94	9.96 (9.75 to 10.18)
Aug.	25188.40	1.82	12833	0.92	-49.05(-49.67 to -48.43)	74897.27	6.81	76423	5.46	2.04 (1.94 to 2.14)
Sept.	21570.60	1.56	18432	1.32	-14.55(-15.03 to -14.09)	70219.19	6.46	75409	5.39	7.39 (7.20 to 7.59)
Oct.	20832.80	1.51	20401	1.46	-2.07(-2.27 to -1.89)	64889.35	5.94	67843	4.85	4.55 (4.39 to 4.71)
Nov.	48485.20	3.51	22783	1.63	-53.01(-53.45 to -52.57)	67718.91	6.18	69640	4.97	2.84 (2.72 to 2.97)
Dec.	301146	21.79	23546	1.68	-92.18(-92.28 to -92.08)	66396.54	6.11	64097	4.58	-3.46(-3.61 to -3.32)
